# Pacemaker Lead Induced Inferior Vena Caval Thrombosis Leading to Portal Hypertension

**DOI:** 10.1016/s0972-6292(16)30609-x

**Published:** 2013-03-07

**Authors:** Sharad Chandra, Deepak Ameta, Sudarshan Kumar Vijay, Sudhanshu Kumar Dwivedi, Ram Kirti Saran

**Affiliations:** Department of Cardiology, King George's Medical University, Lucknow, UP, India

**Keywords:** Pacemaker lead, Inferior vena cava, Thrombosis, Echocardiography

## Abstract

Inferior vena caval thrombosis is an unusual complication of permanent pacemaker implantation. The clinical presentation due to thrombosis depends on the site of thrombus. We have described here a rare case of pacemaker lead associated thrombosis of inferior vena cava, its diagnostic work up and briefly reviewed the existing literature of this uncommon complication.

## Case Report

**Figure 1:**
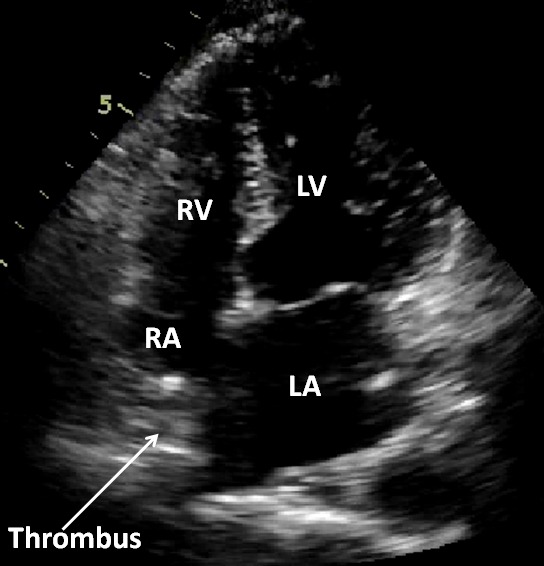
2D Echocardiogram - Apical 4 chamber view showing thrombus in the right atrium

**Figure 2:**
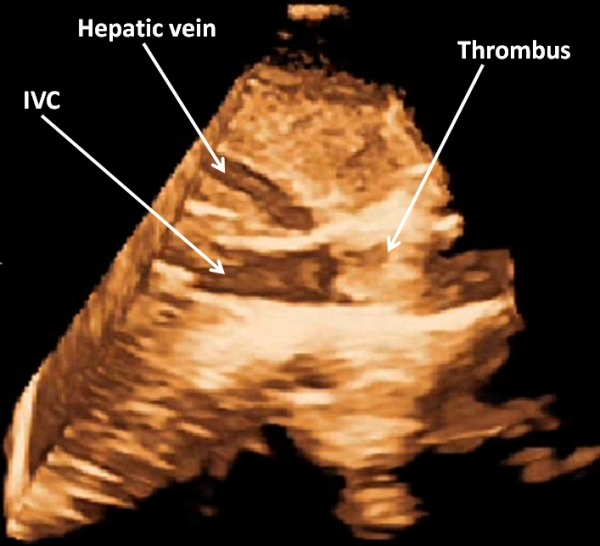
Echocardiogram showing thrombus in the inferior vena cava

**Figure 3:**
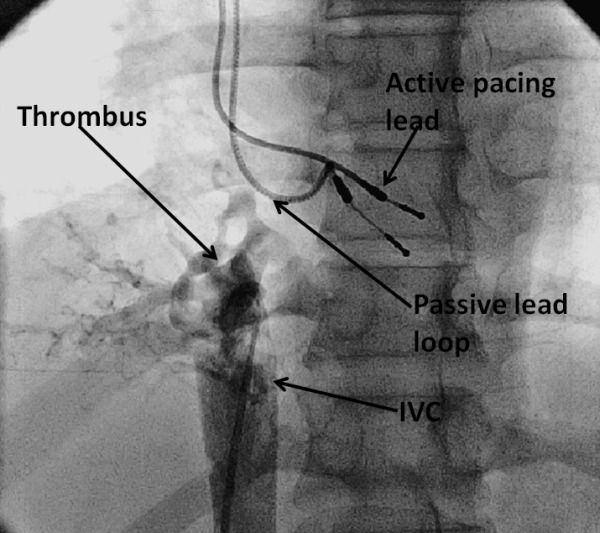
IVC venogram demonstrating no flow from IVC to RA

A 40 year old male had a history of single chamber permanent pacemaker implantation (VVIR) in 2001 for complete heart block. As the patient was young, basic investigations were done at that time to exclude auto - immune and infiltrative diseases. Tests for Anti nuclear antibody (ANA), anti phosoholipid antibodies (APLA) and Rheumatoid factor were negative. In 2008, he was detected to have ERI (Emergency Replacement Indicated) and re-implantation was done with the change of pulse generator and pacing lead as lead impedance was high. The old lead was left in situ. After 4 years, he presented in our department with gradually progressive dyspnoea, bilateral pedal oedema and abdominal distension. At the time of presentation, he was in NYHA II functional class. On examination, the patient had pulse rate of 90/ min, blood pressure of 100/60 and normal heart sounds. General physical examination showed bilateral pedal oedema and icterus. The chest examination was normal. Abdominal examination showed distended superficial abdominal veins flowing centrifugally from umbilicus, tender hepatomegaly and shifting dullness, which was indicative of ascites. ECG showed paced rhythum. Chest radiograph revealed pacemaker in situ with its lead at right ventricular (RV) apex and another functionless lead, also at RV apex. 2D trans-thoracic echocardiography showed normal biventricular functions with paradoxical inter ventricular septal motion. Pacemaker lead was seen across tricuspid valve, positioned at RV apex along with another functionless lead at RV apex. This functionless lead had a small loop in right atrium (RA) close to the opening of inferior vena cava (IVC) into RA. A small mass was seen at this site over the lead with an accompanying thrombus at the mouth of IVC which was completely obliterating it (Figure 1). Similar findings were confirmed with three dimensional echocardiography (Figure 2). Colour Doppler also showed no flow from IVC to RA, instead increased flow was seen from superior vena cava to RA. A huge conglomeration of veins was seen around IVC which were collaterals, diverting blood from IVC to RA. Ultrasonography (USG) of abdomen showed hepatomegaly with dilated hepatic veins, dilated portal vein with a diameter of 12 mm, and moderate ascites. IVC venography showed no flow from IVC to RA, with flow diverting from IVC along collaterals to RA (Figure 3). So, a diagnosis of 'thrombus over the functionless lead along with IVC thrombosis, leading to portal hypertension' was made. Basic laboratory investigations were done for excluding thrombophillic disorders. Levels of Protein C, protein S and antithrombin III were normal; reports for Factor V Leiden mutation and Prothrombin gene mutation were negative. USG abdomen and pelvis did not show any abnormality favouring intra-abdominal malignancy leading to increased thrombogenicity. After this, the patient was put on anti-coagulation. There was no improvement with anti-coagulation and patient was thereafter referred to surgeon for retrieval of thrombus and functionless pacemaker lead.

## Discussion

Pacemakers are life saving devices for the various heart block conditions. However, they are associated with few potential complications. One of them is the venous phlebo-thromboses. Insertion of pacemaker may lead to occlusive or non-occlusive thromboses, mostly involving the leads containing axillary, subclavian veins and superior vena cava. The venography and Doppler confirmed thrombosis of upper veins is relatively high, and may vary from 15- 44% [1].

Sometimes, thrombosis of IVC is also reported. Although, migration of retained pacemaker leads into IVC has been mentioned previously [2], only a few cases of thrombosis of such sites have been reported. Toumbouras et al [3] described such a complication due to migration of a retained functionless pacemaker electrode with subsequent thrombosis of the IVC. Schroeter et al [4] also demonstrated a case of cyanotic congenital heart disease with sick sinus syndrome, which was treated with a permanent pacemaker with subsequent multiple lead replacements. Later, the patient developed complete occlusion of the IVC with stenosis of the SVC with pacemaker leads in both lesions. Also, the patient developed liver failure with ascites and oesophageal varices. Patient was initially treated with a thrombolytic agent and later explantation of the lead was done with stenting of the IVC and SVC lesion.

Lu CL et al [5] also described a case of permanent pacemaker implantation in a patient with refractory paroxysmal supraventricular tachycardia episodes related to Wolff-Parkinson-White syndrome. Three years later, another pacemaker was implanted because of dislodgement of the previous pacemaker. The patient developed symptoms of abdominal pain and ascites 5 years after the second pacemaker implantation. An echocardiogram displayed thrombus formation in the SVC, RA and the inlet of the IVC into the RA. Inferior and superior venacavogram confirmed the above findings. An impression of Budd-Chiari syndrome caused by pacemaker-induced thrombus was made and was managed with removal of the pacemaker, followed by thoracotomy with thrombectomy.

Our report described a unique case of pacemaker lead induced thrombosis of IVC. The functionless lead made a loop in the RA. Initial thrombus formation at this site might be caused by traumatic intimal lesions in RA with formation of thrombus over the lead and gradual progression of the thrombus into the IVC. The venous flow from lower limbs was continued, although compromised, via collaterals to RA, as seen during venography.

This complication emphasized the importance of removal of all functionless pacemaker leads. If that fails, such leads can be removed via surgeries.

If such leads cannot be explanted or if the patient's condition prohibits surgery, such patients should be put on anticoagulation. It is also important that the distal end of pacing lead is very well secured at the site of insertion which completely eliminates the possibility of migration or embolisation [3].
